# Supplementary figures and images for: Efficacy of Mesenchymal-Stromal-Cell-Derived Extracellular Vesicles in Ameliorating Cisplatin Nephrotoxicity, as Modeled Using Three-Dimensional, Gravity-Driven, Two-Layer Tubule-on-a-Chip (3D-MOTIVE Chip)
Source: Int J Mol Sci. 2023 Oct 29;24(21):15726. doi: 10.3390/ijms242115726 (PMC10647511; doi:10.3390/ijms242115726)

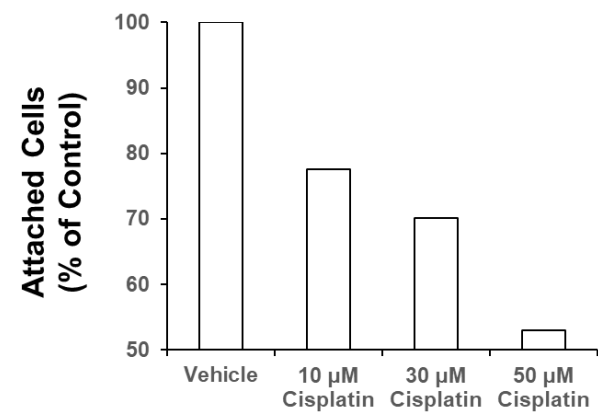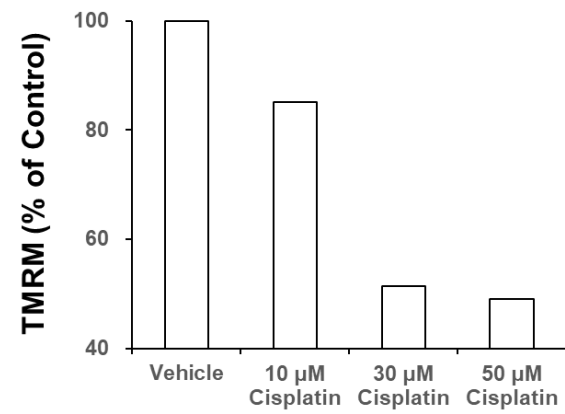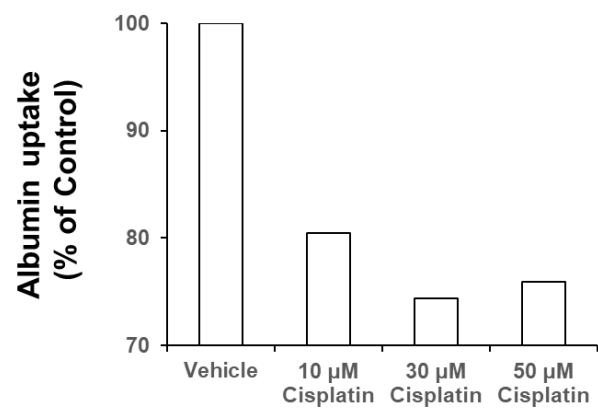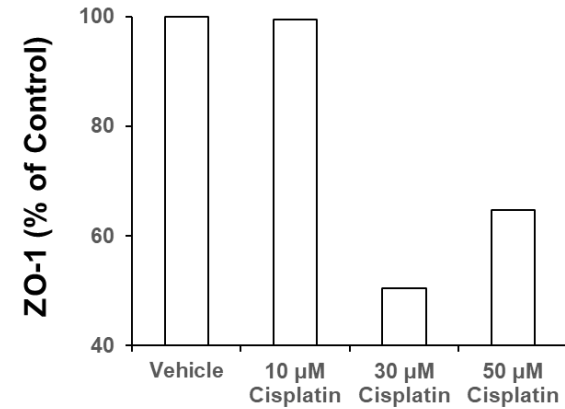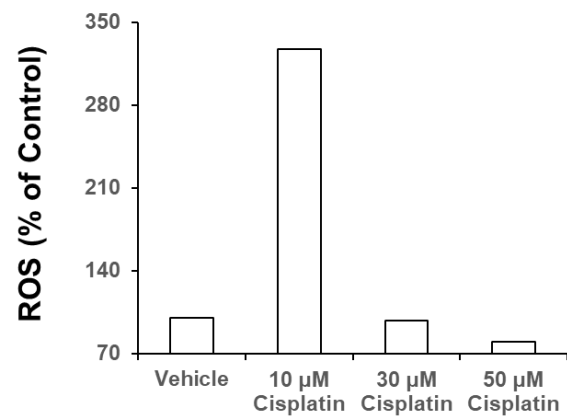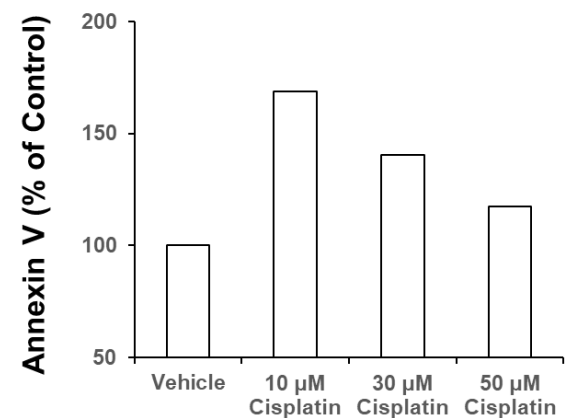

Supplement: Supplementary file 1 [file ijms-24-15726-s001.zip › ijms-2645067-supplementary.pdf]
